# Pseudo-time-reversal symmetry and topological edge states in two-dimensional acoustic crystals

**DOI:** 10.1038/srep32752

**Published:** 2016-09-02

**Authors:** Jun Mei, Zeguo Chen, Ying Wu

**Affiliations:** 1 Department of Physics, South China University of Technology, Guangzhou 510640, China; 2 Division of Computer, Electrical and Mathematical Sciences and Engineering, King Abdullah University of Science and Technology (KAUST), Thuwal 23955-6900, Saudi Arabia

## Abstract

We propose a simple two-dimensional acoustic crystal to realize topologically protected edge states for acoustic waves. The acoustic crystal is composed of a triangular array of core-shell cylinders embedded in a water host. By utilizing the point group symmetry of two doubly degenerate eigenstates at the Γ point, we can construct pseudo-time-reversal symmetry as well as pseudo-spin states in this classical system. We develop an effective Hamiltonian for the associated dispersion bands around the Brillouin zone center, and find the inherent link between the band inversion and the topological phase transition. With numerical simulations, we unambiguously demonstrate the unidirectional propagation of acoustic edge states along the interface between a topologically nontrivial acoustic crystal and a trivial one, and the robustness of the edge states against defects with sharp bends. Our work provides a new design paradigm for manipulating and transporting acoustic waves in a topologically protected manner. Technological applications and devices based on our design are expected in various frequency ranges of interest, spanning from infrasound to ultrasound.

Topological insulators have witnessed a lot of success in the last decade. In various electronic systems, from the artificially designed Haldane lattice^1^, to graphene^2^,^3^ and HgTe/CdTe quantum well structures^4^,^5^, the topology – a mathematical property describing the quantized behavior of the wavefunctions over the associated dispersion bands – has been found to have a profound influence on the transportation properties of electronic wave functions^6^,^7^. The concept of topology was thereafter borrowed from quantum systems and transplanted into classical systems, offering researchers a new degree of freedom in controlling and manipulating electromagnetic^8^,^9^,^10^,^11^,^12^,^13^,^14^,^15^,^16^,^17^,^18^,^19^,^20^,^21^,^22^,^23^,^24^,^25^,^26^,^27^, acoustic^28^,^29^,^30^,^31^,^32^,^33^ and elastic waves^34^,^35^,^36^,^37^,^38^,^39^,^40^,^41^,^42^ in their corresponding artificial structures.

Both electromagnetic and elastic waves satisfy vector wave equations. Electromagnetic wave has two transverse polarizations/modes that are perpendicular to the propagation direction. Elastic wave contains not only two transverse polarizations/modes but also one longitudinal polarization/mode. The polarization degree of freedom is naturally exploited to emulate spins in quantum systems in the realization of unidirectional propagation of classical waves. In contrast, acoustic wave satisfies scalar wave equation and has only one longitudinal polarization, making the realization of unidirectional propagation in acoustics nontrivial. Thus, the reported topological nontrivial phases in acoustic systems were enabled either by rotating fluids to break the time-reversal symmetry^29^,^30^,^31^, or by utilizing chiral interlayer coupling to break the inversion symmetry^32^. Although topologically protected edge states were numerically demonstrated in these works, the need of integrating rotational fluids into resonators or the fabrication of complex inversion-breaking chiral structures remains technically challenging. Very recently, another artificial structure was designed to realize the unidirectional propagation of acoustic waves^33^. The graphite-like structure is a bit complex, which involves two scatterers in each primitive unit cell, and its lattice constant has to be on the wavelength scale to support the associated unidirectional propagation. Here, we propose a different and *simple* way to realize topologically protected edge states for acoustic waves in the *subwavelength* region without introducing rotational fluids or complex unit cells.

The system is a two-dimensional acoustic crystal (AC) composed of a triangular array of core-shell cylinders embedded in a water host. We show that by utilizing the rotational symmetry of the unit cell, pseudo-time-reversal symmetry^23^ can be constructed and, as a result, the acoustic analogue of the quantum spin Hall effect can be emulated. We develop an effective Hamiltonian for the associated system, and unveil the inherent link between the band inversion and the topological phase transition. We unambiguously demonstrate topologically protected acoustic one-way edge states with robust propagation against scattering from defects.

## Results

### Design and characteristics

**Figure 1:**
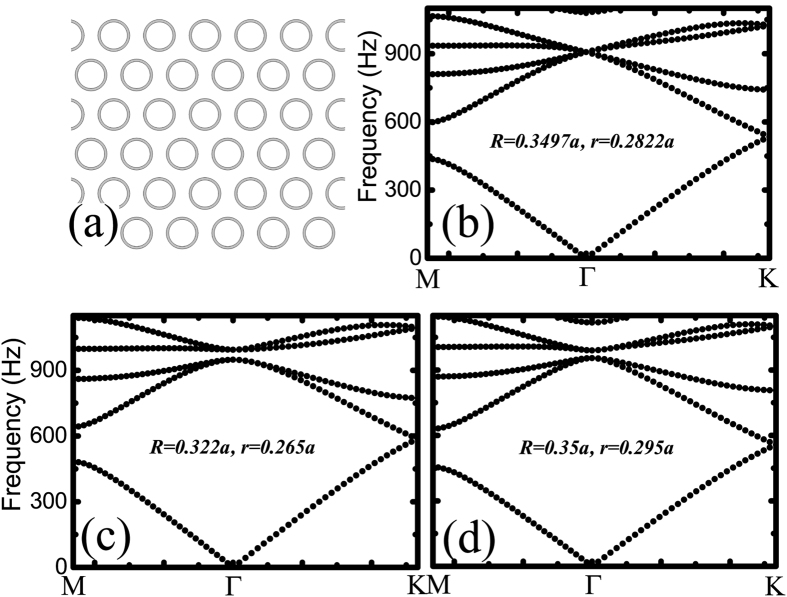
Band structures for the 2D acoustic crystals (ACs). (**a**) Each 2D AC consists of a triangular array of core-shell cylinders embedded in a water host. (**b**) The band structure for *r* = *0.2822a* and *R* = *0.3497a*; a double Dirac cone is seen at the Γ point. (**c**) The band structure for *r* = *0.265a* and *R* = *0.322a*; a trivial gap is formed between the third and fourth bands. (**d**) The band structure for *r* = *0.295a* and *R* = *0.35a*; a nontrivial gap is formed. The frequency range of the gap is almost the same as that in (**c**).

A schematic of the two-dimensional (2D) AC is shown in Fig. 1(a). Each core-shell cylinder has a steel rod with radius *r* as its core, which is coated by a layer of silicone rubber. The outer radius of the cylinder is *R*, and *a* is the lattice constant. The acoustic wave equation is


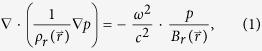


where *p* is the pressure, with *ρ*_*r*_ = *ρ*/*ρ*_0_ and *B*_*r*_ = *B*/*B*_0_ being the relative mass density and bulk modulus, respectively. 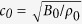 is the speed of sound in water. The mass densities for water, rubber and steel are *ρ*_*0*_ = *1000* *kg/m*^*3*^, *ρ*_*1*_ = *1300* *kg/m*^*3*^, and *ρ*_*2*_ = *7670* *kg/m*^*3*^, respectively. The longitudinal wave velocities in water, rubber and steel are *c*_*0*_ = *1490* *m/s*, *c*_*1*_ = *489.9* *m/s*, and *c*_*2*_ = *6010* *m/s*, respectively. Due to the strong mismatch between the longitudinal velocities in these media, the shear wave modes in the solid components are ignored here, and this simplification does not alter the essential physics of the system^43^,^44^.

It was shown in ref. 44 that a four-fold degeneracy is achieved at the Г point at frequency *ω*_*D*_ = *0.6092(2πc*_*0*_*/a)* when *r* = *0.2822a* and *R* = *0.3497a*, and, as a result, a double Dirac cone was formed at the center of the Brillouin zone as shown in Fig. 1(b). Here, we use COMSOL Multiphysics, a commercial package based on the finite-element method, to calculate the band structures.

The four-fold degeneracy is realized when the doubly degenerate dipolar states coincide with the doubly degenerate quadrupolar states at *ω*_*D*_. The degeneracy is thus accidental. That is to say, if we alter the geometric parameters, e.g., the inner and/or outer radii of the core-shell cylinders, the four-fold degeneracy will be lifted, and the dipolar states will be separated from the quadrupolar states. In Fig. 1(c,d), we plot the band structures for *r* = *0.265a* and *R* = *0.322a*, and for *r* = *0.295a* and *R* = *0.35a*, respectively, where the four-fold degeneracy is lifted and the dipolar states are separated from the quadrupolar states.

**Figure 2:**
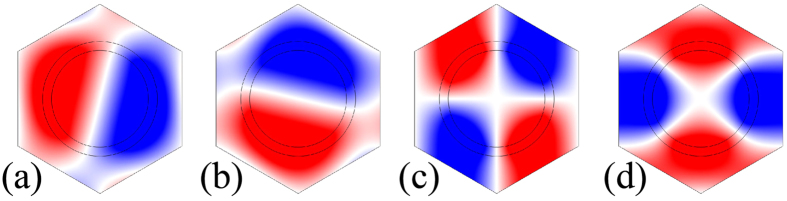
Pressure field distributions of the *E*_*1*_ and *E*_*2*_ representations for the topologically nontrivial AC shown in Fig. 1(d). In (a,b), *p*_*x*_ – and *p*_*y*_ –like pressure fields are seen, while in (c,d) *d*_2*xy*_ – and 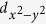 –like patterns are recognized. Red and blue denote the positive and negative maxima, respectively.

For the AC we mentioned above, the point group at the center of the Brillouin zone (BZ) is *C*_*6v*_, which has two 2D irreducible representations^45^: *E*_*1*_ with basis functions (*x,y*) and *E*_*2*_ with basis functions (2*xy*, *x*^*2*^ − *y*^*2*^). *E*_*1*_ modes have odd spatial parity, while *E*_*2*_ modes have even spatial parity. From the distribution of the eigenfields shown in Fig. 2, it is easy to recognize that the *E*_*1*_ [Fig. 2(a,b)] and *E*_*2*_ [Fig. 2(c,d)] representations have, respectively, the same symmetry as the (*p*_*x*_,*p*_*y*_) and 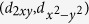 orbitals of electrons in quantum systems. We note that relative eigenfrequencies corresponding to different irreducible representations change as the geometry of the core-shell cylinder changes. To be more specific, the eigenfrequency associated with the *E*_*1*_ representation is lower than that of *E*_*2*_ as shown in Fig. 1(c), but it is higher in Fig. 1(d), indicating that a band inversion process occurs as the geometry changes. Later, we will show that this band inversion is inherently associated with a topological phase transition.

In the Method section, we demonstrate that the spatial symmetry of the *E*_*1*_ and *E*_*2*_ representations can be utilized to construct pseudo-time-reversal symmetry. Here we refer only to the main results. From the *E*_*1*_ basis functions (*p*_*x*_,*p*_*y*_), we can construct the pseudo-spin states as 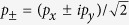, with *p*_+_ (*p*_*−*_) being the pseudo spin-up (spin-down) state. The same conclusion can be made on 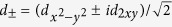, which is another pair of pseudo spin-up/spin-down states associated with the *E*_2_ representation.

### Effective Hamiltonian

To understand the topological property of the band gaps shown in Fig. 1(b,c), we construct an effective Hamiltonian for the current system around the Γ point from a 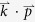 perturbation method^44^,^45^,^46^. We assume Γ_*α*_ (*α* = 1, 2, 3, 4) are the four eigenstates at the Γ point: Γ_1_ = *p*_*x*_, Γ_2_ = *p*_*y*_, 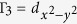, and Γ_4_ = *d*_2*xy*_, with the *p*_*x*(*y*)_ and 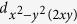 states corresponding to the *E*_*1*_ and *E*_*2*_ representations, respectively. The effective Hamiltonian around the Γ point then is given by 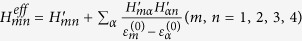, where 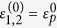 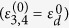 is the eigenfrequency of Γ_1,2_ (Γ_3,4_), and 
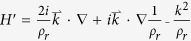
 is the 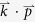 perturbation term for the acoustic wave equation (1), obtained by expanding the Bloch eigenstates at point 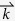 as the linear combinations of the Bloch eigenstates at point Γ. Rewriting the above Hamiltonian on the basis [*p*_+_, *d*_+_, *p*_−_, *d*_−_], we arrive at the following effective Hamiltonian in the vicinity of the Γ point,


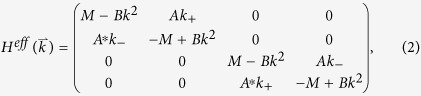


where*k*_±_ = *k*_*x*_ ± *ik*_*y*_ and 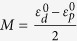 is the frequency difference between *E*_*2*_ and *E*_*1*_ representations at the Γ point, which is positive (negative) before (after) the band inversion. *A* comes from off-diagonal elements of the first-order perturbation term 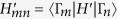 with *m* = 1, 2 and *n* = 3, 4. *B* is determined by the diagonal elements of the second-order perturbation term 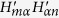, and is typically negative. We note that to derive the above Hamiltonian, the spatial symmetries of the eigenstates, Γ_*α*_, are utilized. The effective Hamiltonian, 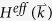, shown in Eq. (2) takes a similar form as that proposed in the Bernevig-Hughes-Zhang (BHZ) model for the CdTe/HgTe/CdTe quantum well system^4^.

We note that, in Fig. 1(c), the bands above (below) the gap belong to the *E*_*2*_ (*E*_*1*_) representation, which means that 
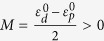
. For the Hamiltonian expressed in Eq. (2), the spin Chern numbers can be evaluated as^4^,^47^


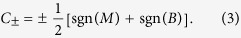


Since C_±_ = 0, we conclude that the band gap shown in Fig. 1(c) is trivial. However the situation is different in Fig. 1(d), where the bands above (below) the gap exhibit *E*_*1*_ (*E*_*2*_) characteristics around the Γ point, meaning that *M* < 0. Applying Eq. (3), we immediately know that C_±_ = ±1 and the gap in Fig. 1(d) is nontrivial. It is evident that the topological property of the effective Hamiltonian is determined by the signs of *M* and *B* rather than their absolute values. It is also interesting to find the topological phase transition from a trivial one [Fig. 1(c)] to a nontrivial one [Fig. 1(d)] that is associated with the band inversion between the *E*_*1*_ and *E*_*2*_ representations around the Γ point. Our finding shares a similar physical mechanism with that in the BHZ model developed for the CdTe/HgTe/CdTe quantum well system^4^. Therefore, we expect that our AC system can support an acoustic ‘spin Hall effect’, although our system is quite different from the BHZ quantum system: we are dealing with a system governed by the classical acoustic wave equation rather than the Schrödinger equation.

### Topological edge states

**Figure 3:**
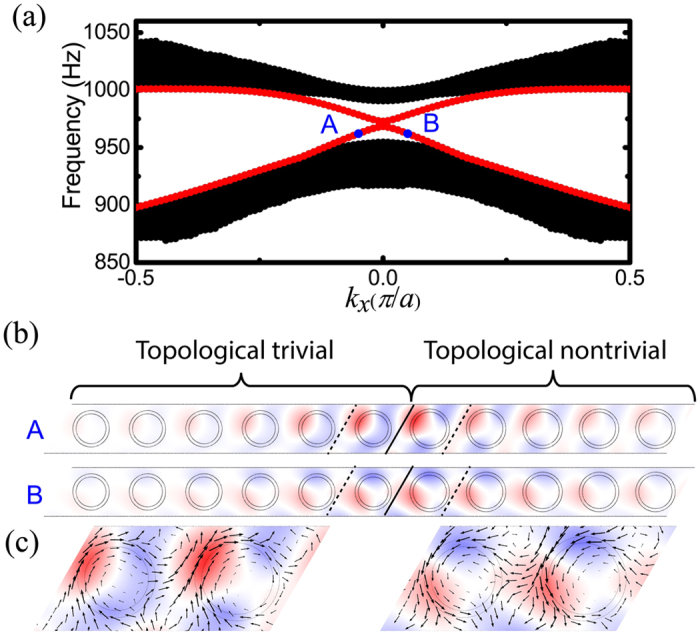
Projected band structure and topological edge states. (**a**) The projected band structure along the Γ*K* direction for a ribbon of topologically nontrivial crystal with its two edges cladded by topologically trivial crystals. The ribbon has 1 unit cell in one direction and 86 unit cells along the other direction (46 nontrivial unit cells cladded by 20 trivial unit cells on both sides). The red and black dots represent edge and bulk states, respectively. (**b**,**c**) Pressure field distributions around the interface between the trivial and nontrivial phases at points A and B, i.e., at *k*_*x*_ = −0.05*π*/*a* and 0.05*π*/*a*, respectively. Red and blue denote positive and negative maxima, respectively. Black arrows indicate the time-averaged Poynting vector.

We consider a ribbon of topologically nontrivial crystal (i.e., the AC that produces the band structure shown in Fig. 1(d)) with its two edges cladded by two topologically trivial crystals (the AC that produces the band structure shown in Fig. 1(c)). The frequency regime, [955.18 Hz, 990.43 Hz], is common for the trivial and nontrivial gaps to create true edge states that are spatially confined around the interface between two crystals. In Fig. 3(a), we plot the projected band structures along the Γ*K* direction for such a ribbon. We find that in addition to the bulk states represented by black dots, there are doubly degenerate states, represented by red dots within the bulk gap region. After examining the eigenfield distributions at the red dots (e.g., points A and B), we find that the pressure field decays exponentially into bulk crystals on both sides, which means that the red curves represent the dispersion relations of edge states that are tightly confined around the interface between the nontrivial and trivial phases. In Fig. 3(b), we plot the pressure field distribution on one interface for the eigenstates at points A and B, respectively, and a magnified view is plotted in Fig. 3(c), where black arrows indicate the time-averaged Poynting vectors. The clockwise and anticlockwise distributions of the Poynting vector at the interface unveil the characteristics of the pseudo spin-up and spin-down states, respectively. The locking of the (pseudo) spin-up and spin-down states with counter-propagations of edge states is reminiscent of the quantum spin Hall effect in electronic systems. We want to note that the working frequency of points A and B is in the subwavelength region, where the lattice constant is only 0.64*λ*_0_.

Because the pseudo-time-reversal symmetry and the pseudo-spin states are constructed on the basis of the *C*_6*ν*_ point group symmetry, any deviation from the crystal symmetry would mix the two pseudo-spin channels as in other topological systems^11^,^15^,^23^. Actually, there is a tiny gap (not evident in Fig. 3(a)) at the Γ point, arising from the reduction of the *C*_6*ν*_ symmetry at the interface between the trivial and nontrivial phases. However, the topological properties of the corresponding structures remain valid even with a moderate deformation in the lattice symmetry, as will be explicitly shown below.

## Discussion

**Figure 4:**
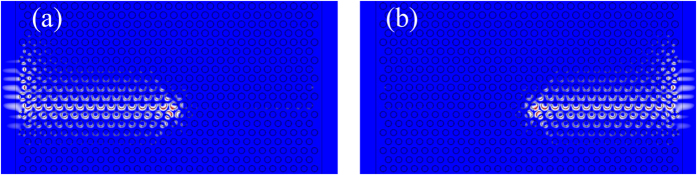
Unidirectional propagation of edge states. Realization of unidirectional acoustic wave propagation towards the (**a**) left and (**b**) right directions along the interface between a nontrivial phase (upper part) and a trivial phase (lower part), with the whole structure surrounded by perfectly matched layers. The acoustic waves that carry the leftward (rightward) wave vector is excited by using a couple of point sources located in the middle part of the interface at a distance of *λ*_0_/4, and the two sources have a phase difference of *π*/2(−*π*/2).

As a first example, we consider a flat edge between a topologically nontrivial phase (upper part) and a trivial phase (lower part), as shown in Fig. 4, where the whole structure is surrounded by perfectly matched layers (PMLs) to absorb outgoing waves. When an acoustic wave carrying a leftward (rightward) wave vector is excited in the middle part of the edge, unidirectional propagation of the acoustic wave towards the left (right) direction can be observed in Fig. 4(a,b). When these edge waves arrive at the left (right) boundary of the simulation domain, they are guided without reflections along the interface of the nontrivial crystal to continue propagating upwards. At the same time, they gradually decay into the PMLs. Negligible reflection occurs at the left (right) boundary of the edge, which is expected from the topological properties of the edge states.

**Figure 5:**
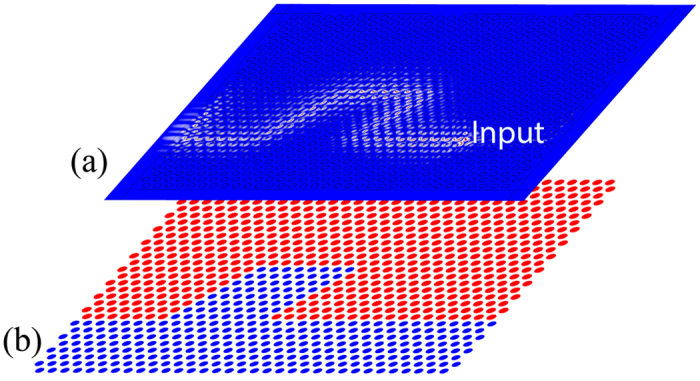
Unidirectional propagation of the edge states along an interface with sharp bends. (**a**) Pressure field distribution. (**b**) A schematic of the sample in which the red dots represent the topologically nontrivial phase while the blue dots indicate the topologically trivial phase.

One of the most important features of topological edge states is that they are immune to defects/imperfections. In the following, we demonstrate edge wave propagation around a specific type of imperfection: four sharp bends of the edge shown in Fig. 5(b), which is constructed from the structure in Fig. 4 by further replacing a region of the nontrivial topological phase 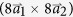 with that of the trivial one. When a left-heading wave is excited, it propagates along the edge and can go around the rhombic defect without reflections at the four sharp corners. It also maintains its unidirectional propagation as shown in Fig. 5(a). This result confirms the topological robustness of the edge states against a sharply curved interface.

To conclude, we have designed a 2D acoustic crystal consisting of a triangular array of core-shell cylinders embedded in a water host. We have shown that a topological phase transition can be obtained by using the band inversion mechanism in such a simple system. A pseudo-time-reversal symmetry can be constructed by utilizing the *C*_6*ν*_ point group symmetry of the *p* and *d* eigenstates at the Γ point, and it follows that pseudo spin-up and spin-down states can be realized in this classical wave system in the same spirit as the quantum spin Hall effect in electronic systems. An effective Hamiltonian is developed for the current acoustic system around the Γ point, and the Hamiltonian unveils the underlying mechanism that links the band inversion to a topological phase transition. Numerical simulations unambiguously demonstrate the unidirectional propagation of the pseudo-spin edge states and the robustness of these edge states against sharp bends. The simple design of our proposed structure suggests that experimental realization is feasible. Since the underlying principle is valid for different scales, we expect that it will have potential applications in manipulating and controlling acoustic waves over a very large frequency range, spanning from infrasound to ultrasound.

## Methods

### Derivation of pseudo-time-reversal symmetry

In the following, we demonstrate that the spatial symmetry of the *E*_*1*_ and *E*_*2*_ representations can be utilized to construct pseudo-time-reversal symmetry^23^. Let D^*E*1^(*C*_6_) and 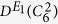 denote the *E*_*1*_ irreducible representations of the *π*/3 and 2*π*/3 rotations, respectively. Their matrix representations on basis (*x*, *y*)^*T*^ are therefore


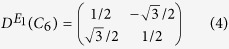



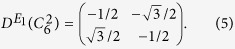


It turns out that they can be combined together in a unitary operator, *U*, as


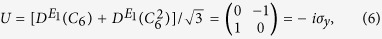


where *σ*_*y*_ is the Pauli matrix. Obviously, *U*^2^ = −*I*. Thus, we can construct an anti-unitary operator, *T*, as *T* = *UK* = −*iσ*_*y*_*K*, where *K* is the complex conjugate operator. It follows that


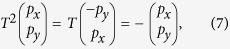


which yields *T*^2^ = −*I*. Similarly, the *E*_2_ matrix representations of rotational operators *C*_6_ and 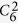 on basis (*x*^2^ − *y*^2^, 2*xy*)^*T*^ are


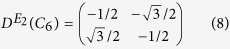



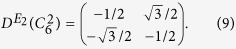


And the unitary operator, *U*, can be constructed as 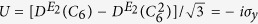. It is easy to check that we also have *T*^2^ = −*I*, the same as in the *E*_1_ irreducible representation. Therefore, for both *E*_*1*_ and *E*_*2*_ modes, the pseudo-time-reversal symmetry, *T* = *UK*, in the current acoustic system indeed satisfies *T*^2^ = −*I*, which is similar to the *real* time-reversal symmetry in electronic systems and which also guarantees the appearance of a Kramers doublet at the Γ point. From the derivations shown above, it is clear that the role played by the crystal symmetry of the unit cell in constructing the pseudo-time-reversal symmetry is crucial.

Following this analysis, we can construct the pseudo-spin states as 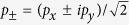, with *p*_+_ (*p*_−_) being the pseudo spin-up (spin-down) state. On the (*p*_+_, *p*_−_)^*T*^ basis, the pseudo-time-reversal operator, *T*′ = *U*′*K*, [*U*′ and *T*′ are defined on the (*p*_+_, *p*_−_)^*T*^ basis, while *U* and *T* are defined on the (*p*_*x*_, *p*_*y*_)^*T*^ basis] exhibits the following desired properties:


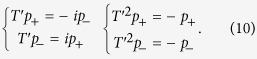


From Eq. (10), it is clear that the wave functions (*p*_+_, *p*_−_) are the two pseudo-spin states in the *E*_1_ representation of our acoustic system because the pseudo-time-reversal operator, *T*′, transforms the pseudo spin-up state into a spin-down state, and vice versa. The same conclusion can be made on 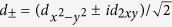, which is another pair of pseudo spin-up/spin-down states associated with the *E*_2_ representation.

### Numerical simulations

All the numerical simulations presented in this article are performed using COMSOL Multiphysics, a commercial package based on the finite-element method. Figures 1, 2 and 3 are computed using the eigenfrequency study in the pressure acoustics module. The Bloch boundary conditions are imposed on the boundaries of the unit cells. Figures 4 and 5 are calculated using the frequency domain study in the pressure acoustics module, where the acoustic crystal structures are surrounded by perfectly matched layers so that there is no reflected wave from the boundaries of the simulation domains.

## Additional Information

**How to cite this article**: Mei, J. *et al*. Pseudo-time-reversal symmetry and topological edge states in two-dimensional acoustic crystals. *Sci. Rep.* **6**, 32752; doi: 10.1038/srep32752 (2016).

## References

[b1] Haldane F. D. M. Model for a Quantum Hall Effect without Landau Levels: Condensed-Matter Realization of the “Parity Anomaly”. Phys. Rev. Lett. 61, 2015–2018 (1988). 10038961 10.1103/PhysRevLett.61.2015

[b2] Kane C. L. & Mele E. J. Quantum Spin Hall Effect in Graphene. Phys. Rev. Lett. 95, 226801 (2005). 16384250 10.1103/PhysRevLett.95.226801

[b3] Hasan M. Z. & Kane C. L. Colloquium: Topological insulators. Rev. Mod. Phys. 82, 3045–3067 (2010).

[b4] Bernevig B. A., Hughes T. L. & Zhang S.-C. Quantum Spin Hall Effect and Topological Phase Transition in HgTe Quantum Wells. Science 314, 1757–1761 (2006). 17170299 10.1126/science.1133734

[b5] Qi X.-L. & Zhang S.-C. Topological insulators and superconductors. Rev. Mod. Phys. 83, 1057–1110 (2011).

[b6] Klitzing K. v., Dorda G. & Pepper M. New Method for High-Accuracy Determination of the Fine-Structure Constant Based on Quantized Hall Resistance. Phys. Rev. Lett. 45, 494–497 (1980).

[b7] Thouless D. J., Kohmoto M., Nightingale M. P. & den Nijs M. Quantized Hall Conductance in a Two-Dimensional Periodic Potential. Phys. Rev. Lett. 49, 405–408 (1982).

[b8] Haldane F. D. M. & Raghu S. Possible Realization of Directional Optical Waveguides in Photonic Crystals with Broken Time-Reversal Symmetry. Phys. Rev. Lett. 100, 013904 (2008). 18232766 10.1103/PhysRevLett.100.013904

[b9] Wang Z., Chong Y. D., Joannopoulos J. D. & Soljăcić M. Reflection-Free One-Way Edge Modes in a Gyromagnetic Photonic Crystal. Phys. Rev. Lett. 100, 013905 (2008). 18232767 10.1103/PhysRevLett.100.013905

[b10] Wang Z., Chong Y., Joannopoulos J. D. & Soljăcić M. Observation of unidirectional backscattering-immune topological electromagnetic states. Nature 461, 772–775 (2009). 19812669 10.1038/nature08293

[b11] Hafezi M., Demler E. A., Lukin M. D. & Taylor J. M. Robust optical delay lines with topological protection. Nat. Phys. 7, 907–912 (2011).

[b12] Poo Y., Wu R.-X., Lin Z., Yang Y. & Chan C. T. Experimental Realization of Self-Guiding Unidirectional Electromagnetic Edge States. Phys. Rev. Lett. 106, 093903 (2011). 21405623 10.1103/PhysRevLett.106.093903

[b13] Fang K., Yu Z. & Fan S. Realizing effective magnetic field for photons by controlling the phase of dynamic modulation. Nat. Photonics 6, 782–787 (2012).

[b14] Rechtsman M. C. . Photonic Floquet topological insulators. Nature 496, 196–200 (2013). 23579677 10.1038/nature12066

[b15] Khanikaev A. B. . Photonic topological insulators. Nat. Mater. 12, 233–239 (2013). 23241532 10.1038/nmat3520

[b16] Hafezi M., Mittal S., Fan J., Migdall A. & Taylor J. M. Imaging topological edge states in silicon photonics. Nat. Photonics 7, 1001–1005 (2013).

[b17] Liang G. Q. & Chong Y. D. Optical Resonator Analog of a Two-Dimensional Topological Insulator. Phys. Rev. Lett. 110, 203904 (2013). 25167412 10.1103/PhysRevLett.110.203904

[b18] Lu L., Joannopoulos J. D. & Soljačić M. Topological photonics. Nat. Photonics 8, 821–829 (2014).

[b19] Chen W. J. . Experimental realization of photonic topological insulator in a uniaxial metacrystal waveguide. Nat. Commun. 5, 5782 (2014). 25517229 10.1038/ncomms6782

[b20] Xiao M., Zhang Z. Q. & Chan C. T. Surface Impedance and Bulk Band Geometric Phases in One-Dimensional Systems. Phys. Rev. X 4, 021017 (2014).

[b21] Ochiai T. Photonic realization of the (2 + 1)-dimensional parity anomaly. Phys. Rev. B 86, 075152 (2012).

[b22] Ma T., Khanikaev A. B., Mousavi S. H. & Shvets G. Guiding Electromagnetic Waves around Sharp Corners: Topologically Protected Photonic Transport in Metawaveguides. Phys. Rev. Lett. 114, 127401 (2015). 25860770 10.1103/PhysRevLett.114.127401

[b23] Wu L.-H. & Hu X. Scheme for Achieving a Topological Photonic Crystal by Using Dielectric Material. Phys. Rev. Lett. 114, 223901 (2015). 26196622 10.1103/PhysRevLett.114.223901

[b24] Estep N. A., Sounas D. L., Soric J. & Alù A. Magnetic-free non-reciprocity and isolation based on parametrically modulated coupled-resonator loops. Nat. Phys. 10, 923–927 (2014).

[b25] Wang Y.-P., Yang W.-L., Hu Y., Xue Z.-Y. & Wu Y. Detecting topological phases of microwave photons in a circuit quantum electrodynamics lattice. npj Quantum Information 2, 16015 (2016).

[b26] Wang Y.-P. . Realizing and characterizing chiral photon flow in a circuit quantum electrodynamics necklace. Sci. Rep. 5, 8352 (2015). 25666884 10.1038/srep08352 PMC4322363

[b27] He C. . Photonic topological insulator with broken time-reversal symmetry. Proc. Natl. Acad. Sci. USA 113, 4924–4928 (2016). 27092005 10.1073/pnas.1525502113 PMC4983855

[b28] Xiao M. . Geometric phase and band inversion in periodic acoustic systems. Nat. Phys. 11, 240–244 (2015).

[b29] Yang Z. . Topological Acoustics. Phys. Rev. Lett. 114, 114301 (2015). 25839273 10.1103/PhysRevLett.114.114301

[b30] Ni X. . Topologically protected one-way edge mode in networks of acoustic resonators with circulating air flow. New J. Phys. 17, 053016 (2015).

[b31] Chen Z. G. & Wu Y. Tunable Topological Phononic Crystals. Phys. Rev. Applied 5, 054021 (2016).

[b32] Xiao M., Chen W. J., He W.-Y. & Chan C. T. Synthetic gauge flux and Weyl points in acoustic systems. Nat. Phys. 11, 920–924 (2015).

[b33] He C. . Acoustic topological insulator and robust one-way sound transport. arXiv:1512.03273 (unpublished).

[b34] Prodan E. & Prodan C. Topological Phonon Modes and Their Role in Dynamic Instability of Microtubules. Phys. Rev. Lett. 103, 248101 (2009). 20366230 10.1103/PhysRevLett.103.248101

[b35] Kane C. L. & Lubensky T. C. Topological boundary modes in isostatic lattices. Nat. Phys. 10, 39–45 (2014).

[b36] Süsstrunk R. & Huber S. D. Observation of phononic helical edge states in a mechanical topological insulator. Science 349, 47–50 (2015). 26138969 10.1126/science.aab0239

[b37] Paulose J., Chen B. G. & Vitelli V. Topological modes bound to dislocations in mechanical metamaterials. Nat. Phys. 11, 153–156 (2015).

[b38] Khanikaev A. B., Fleury R., Mousavi S. H. & Alù A. Topologically robust sound propagation in an angular-momentum-biased graphene-like resonator lattice. Nat. Commun. 6, 8260 (2015). 26440700 10.1038/ncomms9260 PMC4600716

[b39] Mousavi S. H., Khanikaev A. B. & Wang Z. Topologically protected elastic waves in phononic metamaterials. Nat. Commun. 6, 8682 (2015). 26530426 10.1038/ncomms9682 PMC4659837

[b40] Peano V., Brendel C., Schmidt M. & Marquardt F. Topological Phases of Sound and Light. Phys. Rev. X 5, 031011 (2015).

[b41] Wang P., Lu L. & Bertoldi K. Topological Phononic Crystals with One-Way Elastic Edge Waves. Phys. Rev. Lett. 115, 104302 (2015). 26382680 10.1103/PhysRevLett.115.104302

[b42] Swinteck N. . Bulk elastic waves with unidirectional backscattering-immune topological states in a time-dependent superlattice. J. Appl. Phys. 118, 063103 (2015).

[b43] Kafesaki M. & Economou E. N. Multiple-scattering theory for three-dimensional periodic acoustic composites. Phys. Rev. B 60, 11993–12001 (1999).

[b44] Li Y., Wu Y. & Mei J. Double Dirac cones in phononic crystals. Appl. Phys. Lett. 105, 014107 (2014).

[b45] Dresselhaus M. S., Dresselhaus G. & Jorio A. Group Theory: Application to the Physics of Condensed Matter (Springer-Verlag, Heidelberg, Germany, 2008).

[b46] Mei J., Wu Y., Chan C. T. & Zhang Z.-Q. First-principles study of Dirac and Dirac-like cones in phononic and photonic crystals. Phys. Rev. B 86, 035141 (2012).

[b47] Shen S.-Q., Shan W.-Y. & Lu H.-Z. Topological insulator and the Dirac equation. Spin 1, 33–44 (2011).

